# Enhanced Signaling Through the TLR9 Pathway Is Associated With Resistance to HIV-1 Infection in Chinese HIV-1–Exposed Seronegative Individuals

**DOI:** 10.3389/fimmu.2020.01050

**Published:** 2020-05-29

**Authors:** Junjun Jiang, Xi Hu, Wenwei Li, Jie Liu, Bingyu Liang, Hui Chen, Jiegang Huang, Ning Zang, Chuanyi Ning, Yanyan Liao, Rongfeng Chen, Jingzhen Lai, Jiemei Chu, Peijiang Pan, Ping Cui, Qiao Tang, Xiu Chen, Hao Liang, Li Ye

**Affiliations:** ^1^Guangxi Key Laboratory of AIDS Prevention and Treatment, School of Public Health, Guangxi Medical University, Nanning, China; ^2^Guangxi Collaborative Innovation Center for Biomedicine, Life Sciences Institute, Guangxi Medical University, Nanning, China

**Keywords:** HIV-1, HIV-1–exposed seronegative, innate immunity, TLR9, China

## Abstract

Innate immunity is the first line of defense against invading pathogens and may mediate HIV-1 resistance in HIV-1–exposed seronegative (HESN) individuals. This study aims to identify components of innate immunity that confer natural HIV-1 resistance in Chinese HESN individuals. Specifically, we compared the expression levels of Toll-like receptors (TLRs) and associated pathway molecules in peripheral blood mononuclear cells (PBMCs), monocytes/macrophages, and plasma obtained from HESN and control individuals. HESN individuals had higher expression of TLR9, IRF7, IFN-α/β, RANTES, and MIP-1α/1β in PBMCs and plasma than control subjects. Upon TLR9 stimulation, significantly higher expression of TLR9 and IRF7, as well as higher production of IFN-α/β, RANTES, and MIP-1α/1β, was observed in PBMCs and monocytes/macrophages from HESN individuals than in the corresponding cells from control individuals. More importantly, both with and without TLR9 stimulation, the levels of HIV-1 replication in monocyte-derived macrophages (MDMs) from HESN individuals were significantly lower than those in MDMs from control individuals. These data suggest that increased TLR9 activity and subsequent release of antiviral factors contribute to protection against HIV-1 in HESN individuals.

## Introduction

Since the emergence of the global HIV/AIDS epidemic, HIV/AIDS has caused more than 20 million deaths (1). To date, there are no effective medicines to cure HIV/AIDS or vaccines to provide comprehensive protection against infection. Although most individuals become infected after repeated exposure to HIV, epidemiological and clinical studies have identified a unique resistant population (2–4): HIV-1–exposed seronegative (HESN) individuals who are not infected with HIV despite repeated exposure to the virus. Understanding the mechanism of resistance to HIV acquisition in HESN individuals is critical for providing new insight into HIV transmission and would facilitate the development of novel drugs, vaccines, or microbicidal approaches.

HESN individuals have widely been identified among female sex workers (5), intravenous drug users (6), discordant couples (7), children born to HIV-seropositive mothers (8, 9), and homosexual or heterosexual subjects with a history of unprotected sex with their seropositive partners (10). Studies of HESN individuals have revealed several host and viral factors, including genetic, immunological, and sociobehavioral factors, associated with HIV resistance. However, to date, only homozygosity for the CCR5Δ32 mutation (11) has been consistently identified as a mechanism of HIV resistance. However, this mutation accounts for only a minority of cases; in particular, it has rarely been found in the Chinese population. Therefore, the precise mechanisms of protection against HIV in most HESN individuals remain to be determined.

Recently accumulating evidence indicates that the host innate immunity is involved in mediating HIV resistance in HESN individuals. However, the results are inconsistent. Some reports support the hypothesis that enhanced innate immune functions—including increased natural killer (NK) cell numbers (12), heightened dendritic cell responses (13), and increased secretion of antiviral factors such as β-chemokines, small antiviral factors and defensins (14)—provide a resistance mechanism. The results of other studies support the hypothesis that innate immune activation enhances HIV-1 infection and chronic disease progression (15) and that a decrease in innate immune activation contributes to HIV resistance in HESN individuals (16). Understandably, these controversial results were obtained from different studies in different cohorts, in different races, in different human tissues and even with different definitions of HESN (17). To date, few mechanistic studies have focused on Chinese HESN individuals. Thus, identifying whether and how innate immunity is associated with HIV resistance among Chinese HESN individuals is the original aim of the present study.

Pattern recognition receptors (PRRs) play a central role in initiating innate immune activation and bridging innate immunity with adaptive immunity. Numerous PRRs that recognize HIV components have been identified, including various Toll-like receptors (TLRs) (18), retinoic acid-inducible gene-1 (RIG-I)-like receptors, the recently identified DNA-dependent activator of interferon (IFN) regulatory factor, interferon-inducible protein 16 (IFI16) and cyclic GMP-AMP synthase (cGAS) (19). These receptors are among the first-line molecules critical for host defense against invading HIV and are also potent sensors activated for inflammatory reactions (20). In the present study, the differential PRR expression profiles between HESN and control individuals were first screened, and the innate pathway and mechanism(s) involved in mediating HIV resistance in this HESN population were then explored.

## Materials and Methods

### HESN Participants and Healthy Control Subjects

Ten HESN individuals were enrolled at the Center for Disease Control, Guangxi, China. We adopted stringent HESN inclusion criteria as previously described (21). In detail, these HESN individuals were the seronegative partners in discordant couples. These individuals had a history of multiple unprotected sexual encounters with seropositive partners for more than 4 years and at least three acts of unprotected intercourse within the 4 months prior to entry into the study. In addition to the HESN individuals, 10 healthy HIV-1–negative individuals (controls) matched to the 10 HESN individuals in terms of demographic characteristics, including age, sex, ethnicity, and residential location, were enrolled at the Center for Disease Control, Guangxi, China. After enrollment, 12 weeks after the last previous act of unprotected intercourse, the HIV-1–seronegative status of all participants was reconfirmed by enzyme-linked immunosorbent assay (ELISA) and western blotting (WB). None of the subjects included in the study were intravenous drug users or had STIs such as syphilis and other infectious diseases, such as HBV, HCV.

### Ethics Statement

Written informed consent was obtained from each participant prior to enrollment. All procedures were performed in accordance with the relevant guidelines, and all experimental protocols were approved by the Ethics and Human Subjects Committee of Guangxi Medical University (Ethical Review No. 20140305-009).

### Cell Isolation and Cell Culture

Peripheral blood mononuclear cells (PBMCs) were isolated by centrifugation using Ficoll-Hypaque density gradient reagents (GE Healthcare China, Shanghai, China). Monocytes were separated from PBMCs by adherence to plastic culture plates (Corning China, Shanghai, China) in RPMI 1640 medium supplemented with 10% fetal bovine serum, 100 U/mL penicillin, and 100 μg/mL streptomycin. The differentiation of monocytes into macrophages was achieved by culture for 7–10 days in DMEM supplemented with 10% fetal bovine serum, 100 U/mL penicillin, and 100 μg/mL streptomycin.

### PCR-Based Gene Expression Arrays

TLR pathway-focused and IFN-focused gene expression analyses were performed with a human TLR signaling pathway RT-PCR array kit (Qiagen China, Shanghai, China) and a human interferon RT-PCR array kit (Qiagen China, Shanghai, China), respectively. Briefly, total cellular RNA was isolated from PBMCs using a Super Array RT^2^ qPCR-Grade RNA Isolation Kit. The elimination of genomic DNA contamination and first-strand cDNA synthesis were carried out using an RT^2^ First Strand Kit. TLR pathway-focused and IFN-focused gene expression array analyses were performed using RT^2^ Profiler^TM^ PCR array kits. Statistical analysis of data was performed with a data analysis template provided by Qiagen China (Shanghai, China).

### TLR9 Pathway Stimulation and HIV-1 Infection of Cells

For stimulation of the TLR9 pathway, PBMCs or monocyte-derived macrophages (MDMs) were pretreated with the TLR9 agonist ODN2216 (2 μg/mL) or the corresponding control, ODN2243, for 6 h. For HIV infection, HIV-1 BaL was used to infect MDMs. Briefly, macrophages were infected with HIV-1 Bal (p24, 20 ng/10^6^ cells) for 2 h and then cultured for 8 days as previously described (22). The supernatant was harvested at 24 h post-infection for the detection of IFN-α/β, MIP1-α/β, and RANTES by ELISA; cells were harvested for the determination of mRNA expression by qRT-PCR or detection of proteins by flow cytometry. The levels of HIV p24 in the supernatant were determined by ELISA at 12 h, 4 days, and 8 days post-infection.

### Quantitative Real-Time PCR (qRT-PCR)

After treatment, total cellular mRNA was isolated from PBMCs or MDMs using Tri Reagent (Thermo Fisher Scientific China, Shanghai, China). The isolated mRNA was then subjected to reverse transcription using reverse transcriptase (Takara, China). Real-time RT-PCR was performed to detect the mRNA expression of various genes with SYBR Green real-time PCR (Takara China, Dalian, China). The special oligonucleotide primers used in this study were synthesized by the Beijing Genomics Institute (Shenzhen, China), and the sequences are listed in Supplementary Table 1.

### ELISA

The protein levels of IFN-α/β, MIP-1α/1β, and RANTES in plasma or culture supernatants were measured by ELISA kits (Thermo Fisher Scientific China, Shanghai, China). HIV-1 P24 protein levels were measured by an ELISA kit from the Biomedical Engineering Center of Hebei Medical University (Hebei, China).

### Flow Cytometry

Isolated PBMCs were used for flow cytometric detection of the expression of TLR9 and key factors of the TLR9 pathway in monocytes/macrophages. PerCP-Cy™5.5 mouse anti-human CD14 was used to identify monocytes/macrophages. Staining of TLR9 and key pathway factors was performed using the following antibodies: APC rat anti-human TLR9, Alexa Fluor® 647 mouse anti-NF-κB p65, anti-MyD88 (4D6), and PE mouse anti-IRF-7. All antibodies used for flow cytometry were purchased from BD Biosciences, China (Shanghai, China). Flow cytometric data were acquired using a BD FACSCanto II flow cytometer and analyzed with BD FACS Diva software provided by BD Biosciences, China (Shanghai, China).

### Statistical Analysis

If the data follow the normal distribution, the differences between two groups were analyzed using Student's *t*-test. Otherwise, the non-parametric test was applied. To compare the impact on HIV replication by TLR-9 activation among each group, ANOVA analysis was conducted using statistics software SPSS20.0. The data in this study are expressed as the means ± standard deviations (SDs) or the medians ± ranges. Statistical significance was defined as a probability of *p* < 0.05.

## Results

### Characteristics of the Participants

A total of 20 individuals were recruited in this study, 10 of which were HESN individuals with an average of 30 (range, 18–100) reported unprotected sexual contacts per year (data not shown). The other 10 individuals were healthy control subjects. The two groups were comparable in basic demographic characteristics, including age, sex, ethnicity, original residence, and marital status (*p* > 0.05, data not shown). Of the participants, 60% were male and 90% were of Han nationality; the ages ranged from 23 to 48 years (mean, 33.3 ± 8.0 years). All were married or cohabiting with a regular partner.

### Differential Expression Profiles of TLRs and Type I IFN Pathway Molecules in PBMCs From HESN and Control Subjects

**Figure 1:**
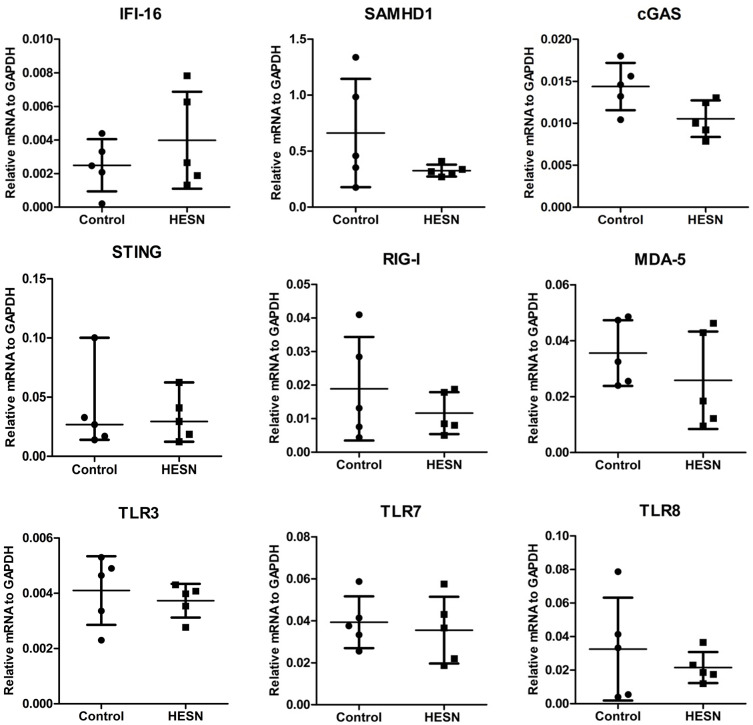
Differential expression profiles of TLRs, PRRs in PBMCs from HESN and control subjects. The expression levels of other HIV-1–recognizing PRRs, including SAMHD1, IFI16, cGAS, STING, RIG-I, MDA-5, TLR3, TLR7, TLR8 in HESN (*n* = 5) and control (*n* = 5) individuals. The mRNA levels were determined by RT-PCR with normalization to the corresponding GAPDH levels and are expressed as relative to the control group. No significant difference was found in the expression of these 9 genes between HESN and control individuals (*p* > 0.05, Student's *t*-test or non-parametric tests).

We first screened the differential expression profiles of various TLRs in PBMCs from HESN and control subjects using a human TLR pathway PCR array. Higher levels of TLR9 and lower levels of TLR10 were found in the HESN subjects than in the controls (Supplementary Figure 1A). However, the expression levels of other TLRs, including those that recognize HIV components (TLR3 and TLR7/8), were similar in the HESN and control subjects (Supplementary Figure 1A). Of the TLR pathway cytokines or related key factors with significant differential expression (increased or decreased by at least 2-fold) between the two groups, most (TNF, NF-κB, IL1a, IL1b, IL2, CCL2, etc.) were upregulated. In particular, a key regulator of the type I IFN pathway, IRF7, was upregulated in the HESN group, implying that the type I IFN pathway may explain the different activation statuses between the HESN and control subjects. Subsequent analysis of the IFN pathway PCR array indicated that, among the significantly differentially expressed (increased or decreased by at least 2-fold) genes in the IFN pathway, most were upregulated (Supplementary Figure 1B). The expression levels of IFN-α and IFN-β exhibited the greatest difference between HESN and control subjects (Supplementary Figure 1B). Because IFN-α/β expression is induced not only by activation of TLR pathways but also by many other HIV-1–recognizing PRRs, including SAMHD1, IFI16, cGAS, STING, RIG-I, MDA-5, TLR3, TLR7, TLR8, we also investigated these expression levels in HESN and control subjects. However, the expression levels of these PRRs (SAMHD1, IFI16, cGAS, STING, RIG-I, MDA-5, TLR3, TLR7, TLR8) in the two groups were not significantly different (Figure 1). Taken together, these data indicate that the TLR9 signaling pathway may be involved in mediating HIV-1 resistance in HESN individuals.

### Increased Expression of TLR9 Pathway Molecules in PBMCs and Monocytes From HESN Individuals

**Figure 2:**
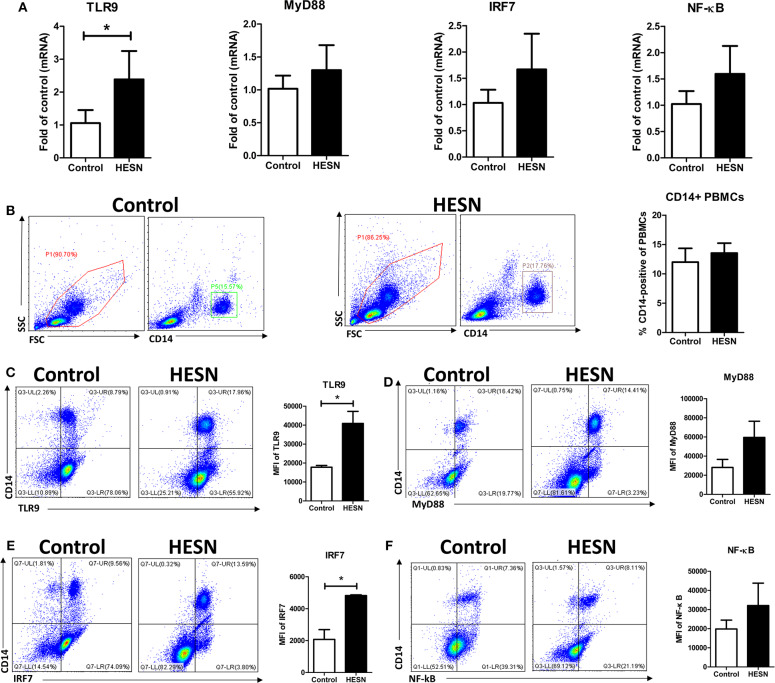
Expression of TLR9 pathway molecules in HESN (*n* = 7) and control (*n* = 7) individuals. **(A)** The mRNA levels of TLR9, MyD88, IRF7, and NF-κB in PBMCs from HESN and control individuals, as determined by RT-PCR with normalization to the corresponding GAPDH levels and expressed as the fold change relative to the control group (which is defined as 1). **(B)** Cytometry dot plots and summary data showing the percentages of monocytes/macrophages (CD14+) in PBMCs from HESN and control individuals. Cytometry dot plots and summary data showing TLR9 **(C)**, MyD88 **(D)**, IRF7 **(E)**, and NF-κB **(F)** expression (mean fluorescence intensity, MFI) in monocytes/macrophages (CD14+) from HESN and control individuals. Summary data are presented as the means ± SDs (*p < 0.05, Student's *t*-test).

To substantiate the protective role of TLR9 in the HESN group, we measured the expression of key genes in the TLR9 signaling pathway: TLR9, MyD88, IRF7, and NF-κB. At the mRNA level, the expression of TLR9 in PBMCs was significantly higher in the HESN group than in the control group (Figure 2A). Although the mRNA levels of MyD88, IRF7, and NF-κB were also higher in the HESN group, the differences were not significant between the two groups (Figure 2A). Both groups had a similar percentage of monocytes (CD14+PBMCs) among PBMCs, indicating that the amounts of monocytes may not be the reason for HIV-1 resistance in HESN individuals (Figure 2B). At the protein level, MyD88 (Figure 2D) and NF-κB (Figure 2F) were higher in HESN but not significantly difference between the two groups, and TLR9 and IRF7 expression levels were significantly higher in monocytes from the HESN group than in the control group (Figures 2C,E), indicating that increased expression of TLR9 pathway molecules in monocytes may be involved in mediating HIV-1 resistance of HESN individuals.

### HESN Individuals Exhibited Increased Levels of Antiviral Factors and CC Chemokines in PBMCs and Plasma

**Figure 3:**
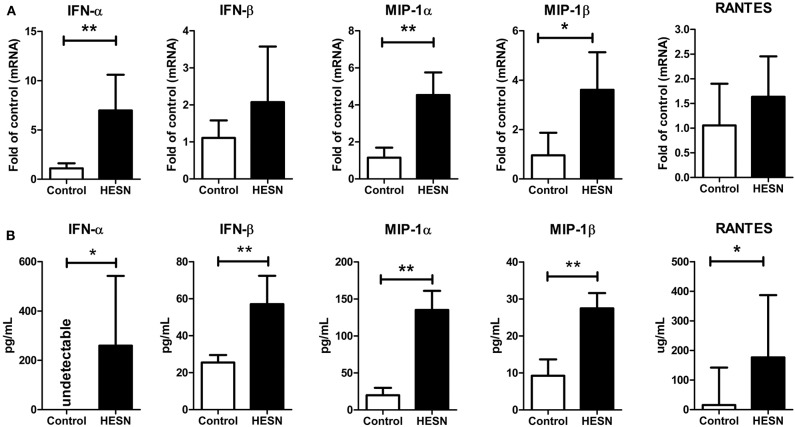
The levels of antiviral factors (IFN-α and IFN-β) and CC chemokines (MIP-1α, MIP-1β, and RANTES) in PBMCs or plasma from HESN (*n* = 10) and control (*n* = 10) individuals. **(A)** The mRNA levels of IFN-α, IFN-β, MIP-1α, MIP-1β, and RANTES in PBMCs from HESN and control individuals were determined by RT-PCR with normalization to the corresponding GAPDH levels and are expressed as the fold change relative to the control group (which is defined as 1). **(B)** The plasma levels of IFN-α, IFN-β, MIP-1α, MIP-1β, and RANTES were determined by ELISAs and are expressed as pg/mL plasma. Summary data are presented as the means ± SDs or the medians ± ranges (*p < 0.05, **p < 0.01; Student's *t*-test or non-parametric tests).

We also investigated the levels of antiviral factors (IFN-α and IFN-β) and CC chemokines (MIP-1α, MIP-1β, and RANTES, the ligands of the HIV entry coreceptor CCR5) related to both the TLR9 pathway and HIV-1 infection/replication. At the mRNA level, HESN individuals exhibited significantly higher expression of IFN-α, MIP-1α, and MIP-1β in PBMCs than control individuals (Figure 3A). At the protein level, the plasma levels of IFN-β, MIP-1α, MIP-1β, and RANTES in HESN individuals were significantly higher than those in control individuals (Figure 3B). In particular, plasma IFN-α was measured at an average level of 259.78 pg/mL in the HESN group but was undetectable in control individuals (Figure 3B).

### Upon TLR9 Stimulation, PBMCs, and Monocytes From HESN Individuals Exhibited Increased TLR9 Pathway Activity

**Figure 4:**
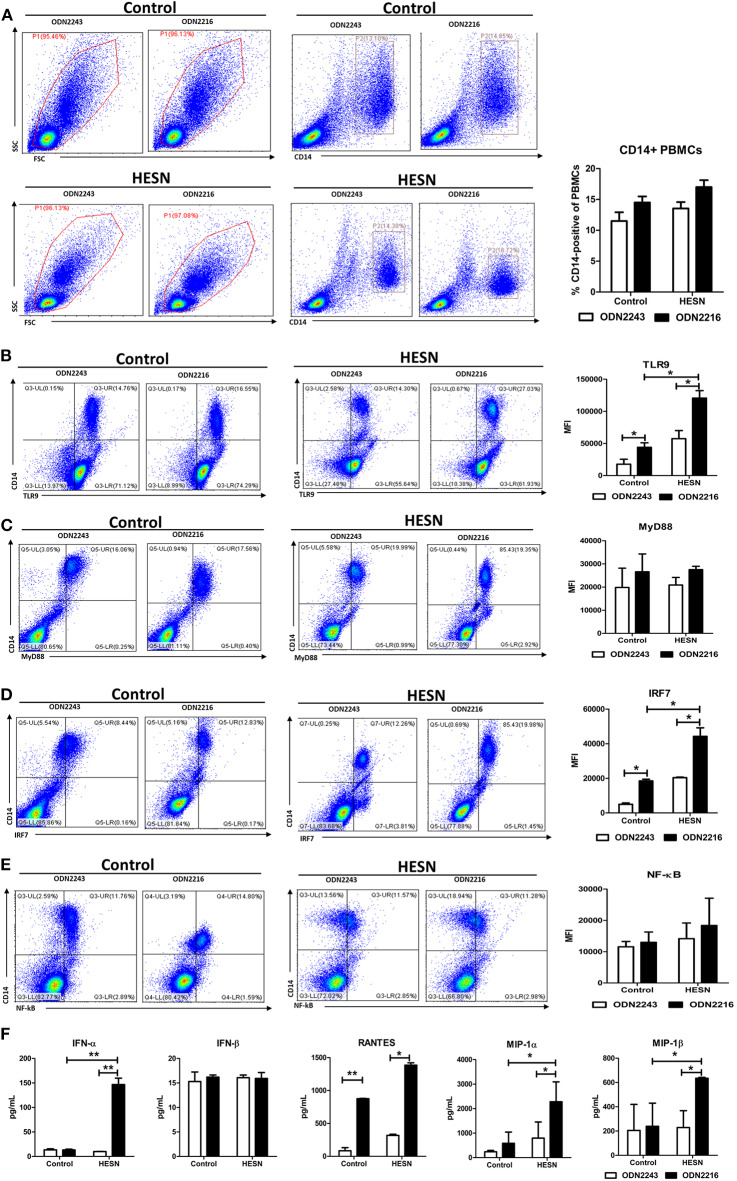
Levels of TLR9 pathway molecules, antiviral factors and CC chemokines in PBMCs from HESN (*n* = 7) and control (*n* = 7) individuals upon TLR9 stimulation. PBMCs isolated from HESN and control individuals were treated with ODN2216 (2 μg/mL) or its corresponding control, ODN2243 (2 μg/mL), for 6 h. **(A)** Cytometry dot plots and summary data showing the percentages of monocytes/macrophages (CD14+) among PBMCs from HESN and control individuals. Cytometry dot plots and summary data showing TLR9 **(B)**, MyD88 **(C)**, IRF7 **(D)**, and NF-κB **(E)** expression (mean fluorescence intensity, MFI) in monocytes/macrophages (CD14+) from HESN and control individuals. **(F)** The levels of IFN-α, IFN-β, RANTES, MIP-1α, and MIP-1β in culture supernatants of PBMCs treated with ODN2216 or ODN2243 were determined by ELISAs. Summary data are presented as the means ± SDs (*p < 0.05, **p < 0.01, ANOVA analysis).

Isolated PBMCs from HESN and control individuals were treated with the TLR9 agonist ODN2216 to measure the functional activity of the TLR9 pathway. TLR9 stimulation slightly increased the percentages of monocytes among PBMCs in both the HESN and control groups, but the differences were not significant either between the two groups or between the groups with and without stimulation (Figure 4A). Upon TLR9 stimulation, ODN2216-treated monocytes from HESN individuals exhibited significantly increased levels of TLR9 (Figure 4B) and IRF7 (Figure 4D) compared with those in monocytes without TLR9 stimulation, an effect that was also observed in control individuals (Figures 4B,D). However, TLR9 stimulation had little effect on the expression of MyD88 (Figure 4C) or NF-κB (Figure 4E) in monocytes in either group.

In the culture supernatants from ODN2216- or ODN2243-treated PBMCs from HESN individuals, TLR9 stimulation significantly enhanced the production of IFN-α, RANTES, MIP-1α, and MIP-1β (Figure 4F). Although *in vitro* stimulation of PBMCs from control individuals also led to the production of RANTES and MIP-1α, stimulated PBMCs from HESN individuals secreted higher total amounts (Figure 4F). However, the production of IFN-β in PBMCs induced by TLR9 stimulation was similar in the two groups (Figure 4F).

### MDMs From HESN Individuals Exhibited Increased TLR9 Pathway Activation

**Figure 5:**
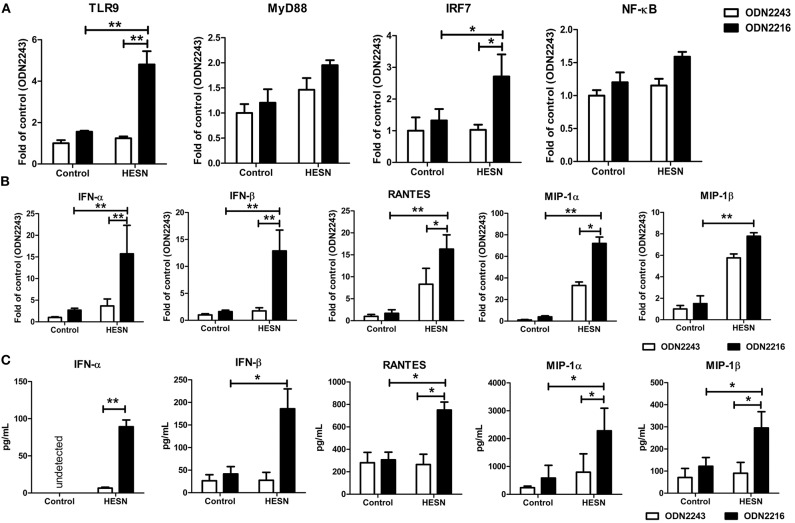
Levels of TLR9 pathway molecules, antiviral factors and CC chemokines in MDMs from HESN (*n* = 7) and control (*n* = 7) individuals upon TLR9 stimulation. Monocytes were separated from PBMCs and cultured for 7–10 days to differentiate into macrophages. MDMs were then treated with ODN2216 (2 μg/mL) or its corresponding control, ODN2243 (2 μg/mL), for 6 h. **(A)** The mRNA levels of TLR9 pathway molecules (TLR9, MyD88, IRF7, and NF-κB) in MDMs were determined by RT-PCR with normalization to the corresponding GAPDH levels and are expressed as the fold change relative to the control group (which is defined as 1). **(B)** The mRNA levels of IFN-α, IFN-β, MIP-1α, MIP-1β, and RANTES in MDMs were determined by RT-PCR with normalization to the corresponding GAPDH levels and are expressed as the fold change relative to the control group (which is defined as 1). **(C)** The protein levels of IFN-α, IFN-β, MIP-1α, MIP-1β, and RANTES in culture supernatants were determined by ELISAs and are expressed as pg/mL supernatant. Summary data are presented as the means ± SDs (*p < 0.05, **p < 0.01; ANOVA analysis).

MDMs from HESN and control individuals were treated with the TLR9 agonist ODN2216 or its corresponding control, ODN2243. Upon TLR9 stimulation, MDMs from HESN individuals exhibited significantly increased mRNA levels of TLR9 and IRF7 compared with those in MDMs from control individuals (Figure 5A). The production of antiviral factors (IFNs and CC chemokines) via TLR9 stimulation in MDMs from HESN individuals was significantly higher than that in control MDMs, as evidenced at both the mRNA (Figure 5B) and the protein level (Figure 5C).

### Lower HIV-1 Susceptibility of MDMs From HESN Individuals Than of MDMs From Control Individuals

**Figure 6:**
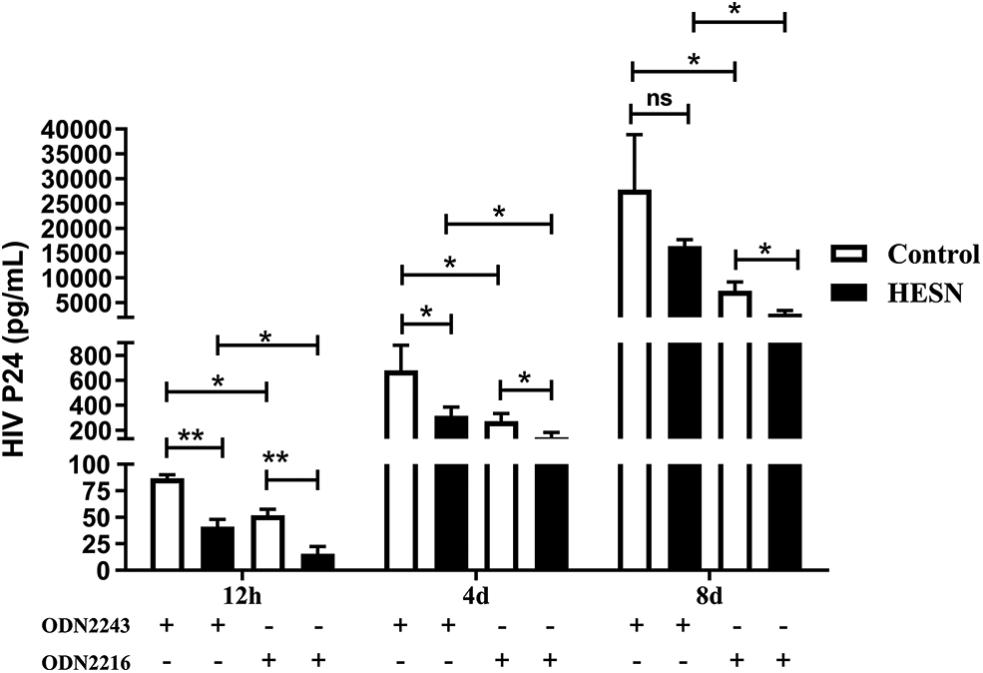
Lower HIV-1 susceptibility of MDMs from HESN individuals (*n* = 7) than of MDMs from control individuals (*n* = 7). Monocytes were separated from PBMCs isolated from HESN or control individuals and cultured for 7–10 days to differentiate into macrophages. MDMs were infected with HIV-1 Bal (p24 20 ng/106 cells) for 2 h and were then treated with ODN2216 (2 μg/mL) or its corresponding control, ODN2243 (2 μg/mL). HIV-1–infected cells were cultured and maintained under treatment for 8 days. The levels of HIV p24 in the supernatant were measured by ELISA at 12 h, 4 days, and 8 days post-infection. Summary data are presented as the means ± SDs (*p < 0.05, **p < 0.01; ANOVA analysis); ns, no significance.

To determine the role of the TLR9 pathway in mediating HIV-1 resistance in HESN individuals, the levels of HIV-1 Bal in MDMs treated with ODN2216 or ODN2243 were compared between the HESN and control groups. MDMs from both HESN and control individuals could be infected with HIV-1 Bal and support HIV-1 replication (Figure 6). However, TLR9 stimulation significantly inhibited HIV-1 replication in both the control group and the HESN group (Figure 6). Importantly, however, both without TLR9 stimulation and with TLR9 stimulation, the levels of HIV-1 replication in the HESN group were significantly lower than those in the control group, indicating that MDMs from HESN individuals are less susceptible to HIV-1 infection than MDMs from control individuals (Figure 6).

## Discussion

The mechanisms involved in mediating natural resistance to HIV-1 infection are multifactorial. The combined contributions of innate and adaptive immunity, as well as genetic factors, provide important advantages that allow a low susceptibility to infection (21, 23–25). Recently, several studies have examined the relationships between host innate immunity and HIV resistance in HESN individuals. Both enhanced and reduced innate immune activation were identified as mechanisms of HIV-1 resistance in certain groups of HESN populations. In the present study, by analyzing innate immunity in Chinese HESNs, we verified that these individuals present a unique activation profile, with increased levels of TLR9 and TLR9 pathway molecules in PBMCs and monocytes/macrophages. We also found that upon TLR9 stimulation, PBMCs and monocytes/macrophages from HESN individuals exhibited increased TLR9 pathway activity, which was associated with the decreased HIV-1 susceptibility of MDMs from HESN individuals. Therefore, the results of our study indicate that Chinese HESN individuals exhibit an immune activation phenotype. It's worth noting that the present study did not include HIV-positive subjects to perform the same analyses, quite a few similar studies in the field also did not include HIV-positive subjects (21, 26–28). The main reason is that HIV infection would make a profound impact on innate immunity, including the TLR-9 pathway. Therefore, the results of our study are mainly come from the comparison between HESN and control healthy individuals.

Typically, monocytes/macrophages are an important component of the innate cellular immune system that recognizes the HIV-1 during HIV-1 infection (29). Macrophages play an important role in the elimination of pathogens, viruses and tumor cells (30) and are differentiated from monocytes under certain conditions (31, 32). In the present study, we chose macrophages as a focus, there are several reasons. Firstly, macrophage is an important innate immune cell, which not only has the strong innate immunity against viral infection but also is the bridge between innate immunity and acquired immunity. Secondly, macrophages are the target cells for HIV infection/replication. Thirdly, as mentioned above, our study aimed to clarify the relationship between innate immunity and HIV susceptibility, it is better to choose innate immune cells for research. Our finding that the increased TLR9 pathway activity in monocytes/macrophages contributes to the decreased HIV-1 susceptibility of MDMs from HESN individuals is consistent with the findings of several previous studies. For example, an earlier study showed that macrophages and activated PBMCs effectively inhibit HIV replication when treated with a TLR9 agonist (33). In addition, a polymorphism in the TLR9 gene is associated with HIV-1 infection, progression, and vertical transmission (21, 34–36). Importantly, the activation of innate immunity through increased TLR9 expression was found in oral epithelial cells from HESN individuals (25). Furthermore, our previous study (22) on drug-abused individuals also shows that decreased TLR9 expression and activity caused by drug abuse, accompanied by the decrease of key factors and cytokines (IRF7, IFN-α/β, etc.) of TLR-9 pathway, contribute to HIV infection/replication in macrophages in drug-abused individuals, which is strong evidence for conclusions of the present study. A limitation of this study we need to point out. Gene expression was only profiled on blood monocytes, but not observed in the *in vitro* derived macrophages. Because the HESN sample source is very limited, the amount of monocyte-derived macrophages can reach even less, which are insufficient for profile arrays. We acknowledged that this is a limitation in our study, after all, many previous studies have highlighted functional differences and HIV susceptibility differences between monocytes and macrophages.

In the present study, Chinese HESN individuals exhibited increased levels of antiviral factors *in vivo*. Moreover, an increased antiviral response (IFN-α/β) induced by TLR9 stimulation was observed in HESN individuals. TLR9 stimulation recruits the downstream adaptor protein MyD88 complex to form the TLR9-MyD88-IRAK-TRAF6 complex, which further activates IRF7, leading to IFN-α/β production (37, 38). IRF7, which is activated by the combination of TLR9 and MyD88 (39, 40), is a core innate immune protein that activates and regulates type I IFNs (41). Type I IFNs (IFN-α/β) enact innate immune antiviral defense (42) and are induced in early HIV-1 infection (43). Indeed, a study revealed that oral epithelial cells exhibit increased expression of IFN-α/β in HESN individuals (25). Moreover, several studies indicated that the TLR9/MyD88/IRF7 signaling pathway may exhibit increased sensitivity to produce type I IFN in order to prevent HIV synthesis, release and replication (37, 44) in HESN subjects than in susceptible subjects. Consistent with this finding, the results of our study indicated that the levels of TLR9, IRF7, and IFN-α/β both *in vivo* and *in vitro* (PBMCs or MDMs activated by the TLR9 agonist) were higher in HESN individuals than in control subjects.

Additionally, we observed that Chinese HESN individuals exhibited higher levels of RANTES, MIP-1α and MIP-1β than control individuals. RANTES is a ligand of CCR5 that inhibits the spread of HIV via binding competitively to the CCR5 receptor to reduce the binding of the HIV envelope glycoprotein gp120 to CCR5 or via inducing the internalization of binding receptors to reduce the surface expression of CCR5 (45). A previous study showed that the production of β-chemokines, including RANTES, was increased in HESN individuals (46). In addition, increased RANTES expression was observed in the genital mucosa of HIV-1–resistant Kenyan commercial sex workers (17). MIP-1α/1β are ligands of CCR5 and play a role in the suppression of HIV replication. Upregulated expression of MIP-1α/1β proteins was found in elite controllers (47), whose MIP-1α/1β levels were sufficient to render susceptible CD4+ T cells resistant to the CCR5-tropic virus. Moreover, an earlier study showed that MIP-1α/1β expression in plasma or genital tract cells was increased in HESN individuals (48). In addition, activated PBMCs were shown to produce high levels of RANTES, MIP-1α and MIP-1β (49, 50).

Taken together, our results suggest that following HIV exposure in Chinese HESN individuals, the increased level of TLR9 expression was associated with enhanced innate immune responses, which, in turn, interfere with HIV replication and productive infection.

## Data Availability Statement

The datasets generated for this study are available on request to the corresponding author.

## Ethics Statement

The studies involving human participants were reviewed and approved by Ethics and Human Subjects Committee of Guangxi Medical University (Ethical Review No. 20140305-009). The patients/participants provided their written informed consent to participate in this study.

## Author Contributions

JJ, LY, and HL conceived and designed the experiments. WL, XH, JLi, QT, and XC: performed the experiments. WL, JJ, XH, BL, JH, and JC: analyzed and interpreted the data. HC, NZ, PC, CN, YL, PP, JLa, and RC: contributed reagents, materials, and analysis tools. XH, JJ, WL, LY, and HL: contributed to the writing of the manuscript.

## Conflict of Interest

The authors declare that the research was conducted in the absence of any commercial or financial relationships that could be construed as a potential conflict of interest.
