# Supplementary material for: Enhanced Signaling Through the TLR9 Pathway Is Associated With Resistance to HIV-1 Infection in Chinese HIV-1–Exposed Seronegative Individuals
Source: Front Immunol. 2020 May 29;11:1050. doi: 10.3389/fimmu.2020.01050 (PMC7274031; doi:10.3389/fimmu.2020.01050)
Supplement: Supplementary file 1 [file Data_Sheet_1.docx]

Table S1. Primers used for real-time RT-PCR in this study.

Supplementary Table 1. Primers used for real-time RT-PCR in this study.

| Gene | Orientation | | Sequence |
| --- | --- | --- | --- |
| GAPDH | | Sense | 5’-GGTGGTCTCCTCTGACTTCAACA-3’ |
|  |  | Antisense | 5’-GTTGCTGTAGCCAAATTCGTTGT-3’ |
| TLR9 | | Sense | 5’-TACCAACATCCTGATGCTAGACTC-3’ |
|  |  | Antisense | 5’-TAGGACAACAGCAGATACTCCAGG-3’ |
| MyD88 | | Sense | 5’-TGGGTCCTTTCCAGAGTTTG-3’ |
|  |  | Antisense | 5’-GCACATGGGCACATACAGAC-3’ |
| IRF7 | | Sense | 5’-TGGTCCTGGTGAAGCTGGAA-3’ |
|  |  | Antisense | 5’-GATGTCGTCATAGAGGCTGTTGG-3’ |
| RANTES | | Sense | 5’-AGCTACTCGGGAGGCTAAGG-3’ |
|  |  | Antisense | 5’-GAGGCATGCTGACTTCCTTC-3’ |
| NF-κB | | Sense | 5’-CTGAACCAGGGCATACCTGT-3’ |
|  |  | Antisense | 5’-GAGAAGTCCATGTCCGCAAT-3’ |
| IFN-α | | Sense | 5’-TTTCTCCTGCCTGAAGGACAG-3’ |
|  |  | Antisense | 5’-GCTCATGATTTCTGCTCTGACA-3’ |
| IFN-β | | Sense | 5’-GCCGCATTGACCATCTATGAGA-3’ |
|  |  | Antisense | 5’-GAGATCTTCAGTTTCGGAGGTAAC-3’ |
| MIP-1α | | Sense | 5’-GCTGACTACTTTGAGACGAGC-3’ |
|  |  | Antisense | 5’-CCAGTCCATAGAAGAGGTAGC-3’ |
| MIP-1β | | Sense | 5’-CCAAACCAAAAGAAGCAAGC-3’ |
|  |  | Antisense | 5’-AGAAACAGTGACAGTGGACC-3’ |
| RIG-I | | Sense | 5’-CTTGGCATGTTACACAGCTGAC-3’ |
|  |  | Antisense | 5’-GCTTGGGATGTGGTCTACTCA-3’ |
| MDA-5 | | Sense | 5’-ACATAACAGCAACATGGGCAGTG-3’ |
|  |  | Antisense | 5’-TTTGGTAAGGCCTGAGCTGGAG-3’ |
| cGAS | | Sense | 5’-CCCAAGCATGCAAAGGAAGG-3’ |
|  |  | Antisense | 5’-ACAATCTTTCCTGCAACATTTCT-3’ |
| STING | | Sense | 5’-CACCTGTGTCCTGGAGTACG-3’ |
|  |  | Antisense | 5’-CATCTGCAGGTTCCTGGTAGG-3’ |
| SAMHD1 | | Sense | 5’-TGTAAGACTGCCCCCAACAG-3’ |
|  |  | Antisense | 5’-ATAACATCGCCATCCTGCGG-3’ |
| IFI16 | | Sense | 5’-CTCCTGGAGCTCAGAACCCG-3’ |
|  |  | Antisense | 5’-AGGAGTCCGAAGATGGCTCT-3’ |
| TLR3 | | Sense | 5’-TTGCCTTGTATCTACTTTTGGGG-3’ |
|  |  | Antisense | 5’-TCAACACTGTTATGTTTGTGGGT-3’ |
| TLR7 | | Sense | 5’-TCCTTGGGGCTAGATGGTTTC-3’ |
|  |  | Antisense | 5’-TCCACGATCACATGGTTCTTTG-3’ |
| TLR8 | | Sense | 5’-ATGTTCCTTCAGTCGTCAATGC-3’ |
|  |  | Antisense | 5’-TTGCTGCACTCTGCAATAACT-3’ |

Fig. S1 Differential expression profiles of TLRs, PRRs in PBMCs from HESN and control subjects

**B**

**A**


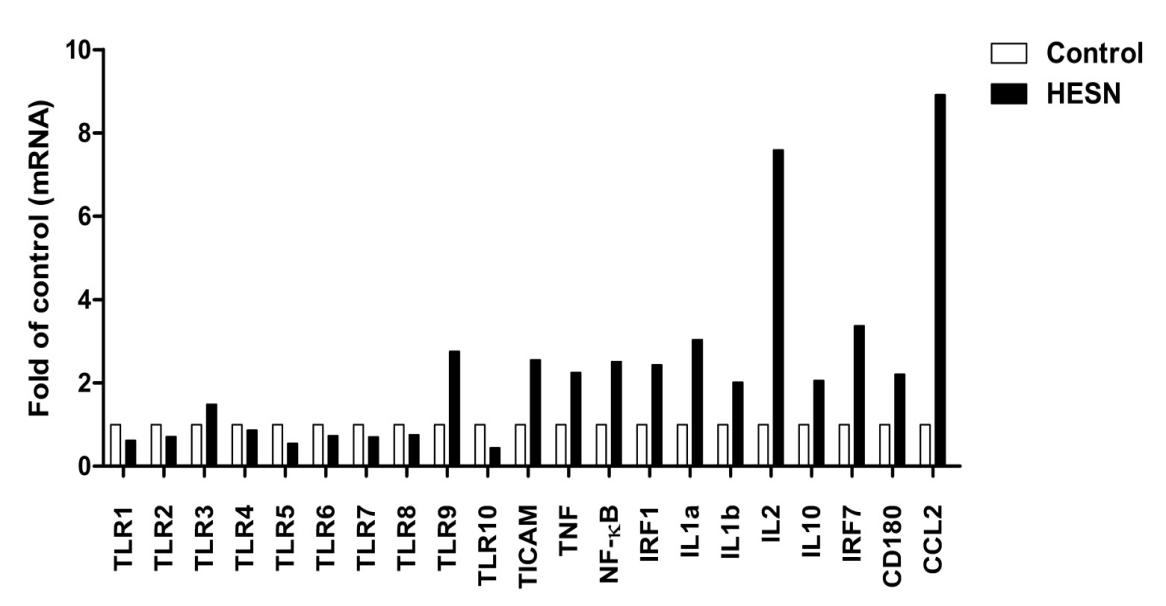

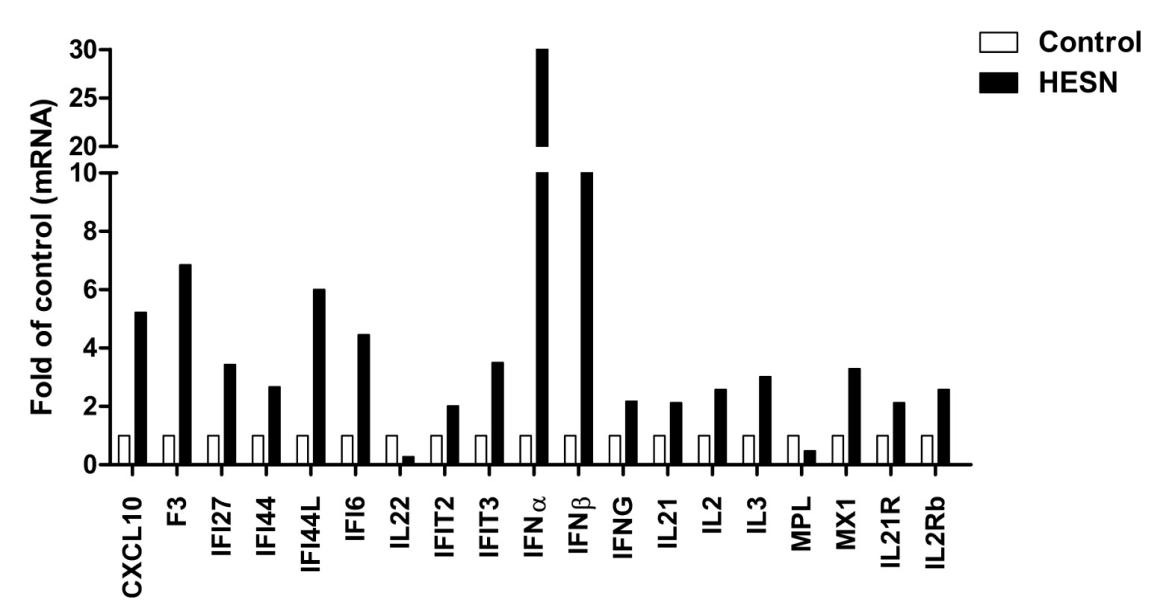


Supplementary Fig. 1 Differential expression profiles of TLRs, PRRs in PBMCs from HESN and control subjects. (A) The expression levels of 10 human TLRs as well as the differential expression of genes in the TLR pathway (HESN vs. Control, fold change of at least 2-fold) between HESN (n=1) and healthy control (n=1) individuals were measured using a TLR pathway PCR array kit. The data are expressed as the fold change relative to the control value, which is defined as 1. (B) The differential expression of genes in the IFN pathway between HESN (n=1) and healthy control (n=1) individuals was measured using an IFN pathway PCR array kit. The data are expressed as the fold change relative to the control value, which is defined as 1.
